# Medical Intelligence

**Published:** 1822-06-01

**Authors:** 


### MEDICAL INTELLIGENCE.

"Sparsa Colligens."

### 1. ROYAL COLLEGE OF PHYSICIANS.

This venerable institution, founded by Linacre, who first brought antient letters into this country,* chartered by our Eighth Henry—and endowed by the immortal Harvey, after he had expounded his splendid doctrine of the circulation within its precincts, is, we are happy to learn, about to be translated from the smoke and fog, and gloomy solitude of Warwick Lane, to the "verge of the palaces," and the clearer atmosphere of Pall-Mall-East. The Crown has bestowed a piece of ground on the College whereon to erect an edifice worthy of the British Metropolis, and of the most dignified and enlightened faculty of medicine in Europe. His Majesty has, moreover, ordained, that the president of the college shall, in future, hold, *ex officio*, the rank and situation of physician in ordinary to His Majesty's person—a distinction, as may be readily perceived, of the utmost importance to the college, and likely to prove a stimulus throughout every ramification of its members. Of the terms in which this mark of Royal favour was conveyed, (in the hand writing we have heard of the King himself,) some idea may be gathered from the following official and gazetted answer to His Majesty's gracious communication.

"To the King's most excellent Majesty.

"Sire,

"We, the President, Elects, and Fellows of the Royal College of Physicians, humbly approach your Majesty with the most grateful acknowledgments for the mark of Royal favour with which your Majesty has been pleased to distinguish us, by an order written and signed by your Royal hand, addressed to Sir Henry Halford, Bart. our present president, commanding him to declare to the College assembled, your Majesty's Royal will and pleasure, that every future president of the College of Physicians, for the time being, shall hold the office of one of your Majesty's physicians in ordinary.

"We associate, Sire, with this mark of your Royal kindness, the pleasing remembrance of the circumstances of our original foundation by your Majesty's illustrious predecessor, King Henry VIII. and dare to presume, from so gracious a proof of your confidence in us, that your Majesty entertains a favourable opinion of our institu-

* "Thomas Lynacrus, Regis Henrici VIII. Medicus; vir et Græcè et Latinè, atque in re medica longe eruditissimus. . . . . . In hæc urbe Collegium Medicorum fieri sua industria curavit, cujus et præsidens proximus electus est, &c."—*Epitaph written by Dr. Caius.*

tions and discipline, as well calculated to make our profession respectable in this country, above what it is in any other part of Europe, and most capable of forming a physician, worthy to be placed near the sacred person of the King.

To our president, Sire, we entrust this expression of our dutiful thanks, our loyalty, our attachment, and devotion to your Majesty; and we pray that no weight of cares which your Majesty's great office imposes upon you, may prove injurious to your health, and that Providence, in his infinite goodness, may continue to watch over a life so highly important to the welfare and happiness of your kingdoms."

We rejoice exceedingly to see Royal favours descend on the Colleges of Physicians and Surgeons in this country. It requires but little acquaintance with men and things to perceive, that honours or dignities conferred by a sovereign or legislature, whether on public bodies or private individuals, prove powerful inducements to those actions and that conduct which maintain or ennoble the dignities so conferred. To be distinguished by *others*, and especially our superiors, is no mean stimulus to the effort still farther to distinguish *ourselves.*

We profess ourselves to be friendly to those institutions which prescribe a regular and even a difficult path to the SUMMI HONORES in medical science. The dreadful effects which have resulted in France, from rendering physic and surgery accessible to the very dregs of society, are well known; and we have only to refer to the oration of M. Richerand himself, before the faculty of medicine of Paris,* for confirmation. We sincerely hope, therefore, that the colleges at the head of medicine and surgery, in Great Britain, will zealously guard the portals of science from the intrusion of those whose qualifications or moral character might sully the lustre of a profession now standing deservedly high in the estimation of all classes of society in this country. The rank, talents, and liberality of those illustrious individuals who at present preside over the institutions in question, are sufficient guarantees for that faithful and honorable discharge of their high and important functions which may at once reflect lustre on themselves and confer a benefit on mankind.

---

2. *Nux Vomica.* A patient had a paralytic affection of one side for some years. The nux vomica (in doses of 25 grains of the powder, three in the 24 hours) was given by Dr. Birkbeck, with the effect of producing violent contractions of a painful and involuntary nature, in the muscles previously paralyzed. The dose was therefore reduced to fifteen grains, which agreed well with the patient, and the paralysis was apparently cured.

Many years ago, Dr. Marcet treated a patient in Guy's Hospital

---

* See No. 4 of this series, p. 771.

(who had been paraplegic for several years) with the nux vomica. The patient so far recovered under this treatment, as to be able to walk about tolerably well. The same remedy, however, was tried by the same physician in two or three other instances of a similar kind, but without success.

Dr. Baillie has remarked that paraplegia is perhaps very often dependent on disease in the brain. This remark has been confirmed, or at least corroborated, by some cases of the kind which fell under the observation of Mr. Henry Earle, in which there were, from the beginning, some affections of certain nerves of sense, originating necessarily in the brain, and where, in process of time, the upper extremities became paralytic, and finally dissection shewed the seat of disease to be in the brain.

---

3. *Cubebs and Capivi.* We have found a combination of these two medicines very useful in the earliest stage of gonorrhœa. Say an ounce of the capivi and four drachms of the cubebs powder, in an eight ounce emulsion to be taken in two days.

---

4. *Sloughing Chancre.* A gentleman had a sloughing chancre — that is, a chancrous ulceration assuming a sloughing appearance, and rapidly spreading over the glans penis. In 48 hours one-third of the glans was destroyed. Local bleeding, purging, fomentations, and the strictest antiphlogistic measures had no effect in arresting the alarming progress of the disease. An eminent surgeon saw the patient. He advised a rapid introduction of mercury, so as to bring on ptyalism as quickly as possible. By extensive frictions, the mouth was made sore in 36 hours. That instant the sloughing ulteration ceased, as if arrested by a charm. We know this treatment is not according to orthodox canons; but it has, since the above case, been tried in several others, and with similar success.

---

5. *Crying of the Fœtus in Utero.* This curious physiological fact (if it be one) is as well authenticated as any thing supported on human testimony—at least medical testimony, can be. It has been recently conveyed with its documentary evidence, to one of the first physicians in London, and by him communicated to the Medico-Chirurgical Society. The circumstance took place in Prussia. A lady, during pregnancy, had experienced some distresses of mind, and had had several discharges of the liquor amnii. In the eighth month of pregnancy while in bed; and while several of her friends and relations were supping in her bed-room, the cries of a child were distinctly heard by all present, under the bed-clothes. The midwife being one of the party, and thinking that the child was suddenly born, desired the company to leave the room immediately, and the physician, who was in the house, to be summoned up. The physician was in time to hear the cries also, which were now unequivocally distinct, in consequence of the bed-clothes being raised. The os uteri was examined, but no dilatation had yet taken place. The cries were now several times reiterated, and then ceased. Labour came on a few hours afterwards, and the child was delivered; a considerable quantity of liquor amnii following the expulsion of the fœtus. The infant was very weak, and died a few hours afterwards.

An explanation has been attempted on the supposition that when the waters escaped, previously to this extrordinary event, air had entered the cavity of the uterus, and thus enabled the fœtus to exercise its vocal powers at a premature period. But such explanation is invalidated by the considerable discharge of liquor amnii at the birth of the child. In fine, notwithstanding the circumstantial evidence and the respectability of the parties concerned, we are forced to withhold our belief of the fact, or rather of the assertion above detailed.

---

6. *Laurel Water.* This medicine, which is far more certain in its effects and imperishable in its nature than prussic acid, is coming into use in the Metropolis, as it has long been on the Continent, in febrile and inflammatory affections, where farther bleeding is deemed unsafe. It has also been successfully given in puerperal fever. It is made by distilling two drachms of the fresh laurel leaves chopped, with four ounces of water, recommitting the distilled water twice afterwards on the same quantity of fresh leaves, and making ultimately four ounces of the menstruum. From thirty to sixty minims of this is given every four or six hours, till a completely sedative effect is produced on the system, and the febrile and inflammatory symptoms are subdued.

A physician-accoucheur of considerable practice in this metropolis, has, for nearly two years past, been in the habit of treating puerperal fever, after one or two copious bleedings, with digitalis in large doses—two or three grains every three or four hours, till the increased action of the vascular system is completely quelled. We recommend a trial of this plan.

---

7. *The late Dr. Parry.* The following medical gentleman *personally* attended the funeral of the late Dr. Parry, and subscribed to erect a monument to his memory. Doctors—Sir G. Gibbs, Haygarth, Crawford, Davis, Barlow, Muttleburry, Robertson, Langworthy, Fisher, Mogg. Surgeons—Tudor, Norman, Day, Geo. Norman, Soden, Brown, Carn, Kitson, Hay, Plinn, Pendrill, Combs, Turner, Roe, Spry, Goldstone, Crook, Anderson, Long, King, Sloper, Mayhew, Crosbie, Williams, Grant, Walker, Gray, G. Goldstone, Godfrey, White, Day.

---

8. *A simple Argument not always inconclusive.* A learned and ingenious cotemporary (in the Quarterly Journal of Foreign Medicine) has laboured hard to prove the non-existence not only of spirit but of MATTER also. "We flatly and fearlessly deny the existence of a certain thing which is called *matter*, and another certain thing which is called *spirit*, and we call for the proof of their existence from those who use the names, and who talk about what we affirm to be *nonentities*."* A young gentleman, on reading this passage, observed to a brother student that, "as for the existence of *spirit*, he could not say; but if the learned editor had been at St. Ge[illegible] Hospital that morning, he would have seen nearly a pint of *matter* discharged by Mr. Gunning from a large abscess."

---

9. *Vapour Baths.* Mr. Seaman, (5, Downing Street, Westminster) has fitted up the humid sulphur vapour, and other baths, at his residence in Downing Street, and we can safely recommend them wherever they are deemed adviseable by the medical attendant.

---

10. *Widows' Fund of Army Medical Officers.*

The anniversary of this most excellent Institution was celebrated on the 15th of May, by a public dinner at the Thatched-House Tavern, where nearly one hundred gentlemen sat down to a sumptuous repast. Nothing could exceed the harmony, order, hilarity, and decorum, that prevailed. The philanthropic principle that first gave origin to the institution, seemed to pervade the whole of this assemblage—a principle, by the bye, which surely does honor to human nature, since it induces us to make sacrifices in *this world*, the benefits accruing from which, can only be enjoyed, *and that by others*, when we are in *another world*.

Among the distinguished professional characters which we observed on both sides of the president, (Dr. Ferguson,) were, Sir Henry Halford, Dr. Baillie, Sir James M'Grigor, Sir Gilbert Blane, Sir E. Home, Sir M. Tierney, Dr. Cooke, Dr. Chambers, Mr. Keate, Mr. Guthrie, Dr. Gordon, Dr. Burnett, Mr. Brookes, Inspector Gunning, Dr. Borland, Dr. Uwins, Dr. Forbes, Dr. Vetch, Dr. Gordon Smith, cum multis aliis.

---

11. On the 6th February, was held the Third Anniversary of the Hunterian Society, when the following Members were elected Officers for the ensuing Year.—*President*, Benjamin Robinson, M.D. —*Vice-Presidents*, William Babington, M.D. F.R.S. B. C. Pierce, M.D. Thomas Callaway, Esq. John Dunston, Esq.—*Treasurer*, B. Robinson, M.D.—*Secretaries*, J. T. Conquest, M.D. William Cooke, Esq.—*Council*, Sir William Blizard, F.R.S. H. Greenwood, Esq. Z. Newington, Esq. J. C. Knight, Esq. R. Dunglison, Esq. J. Miles, Esq. J. Roberts, Esq. Eusebius A. Lloyd, Esq. M. Gosset, Esq. H. Hawkins, Esq. H. Johnson, Esq. and J. Leadham, Esq.—And on the following day, the Members and their Friends dined together at the London Tavern, on which occasion, in consequence of the unavoidable absence of the President, Dr. Babington took the Chair.

---

* Quarterly Journal for April 1822, p. 258.

